# Supplementary material for: Effects of Prospective Audit and Feedback in Patients with Extended-Spectrum β-Lactamase-Producing Escherichia coli Bacteremia
Source: Microorganisms. 2024 Nov 9;12(11):2275. doi: 10.3390/microorganisms12112275 (PMC11596308; doi:10.3390/microorganisms12112275)
Supplement: Supplementary file 1 [file microorganisms-12-02275-s001.zip › microorganisms-3295924-supplementary.pdf]

Table S1. Factors associated with de-escalation from broad-spectrum to CMZ (univariate and multivariate analyses).

Variables		No de-escalation to CMZ (n = 26)	De-escalation to CMZ (n = 36)	*P* value	Multivariate analysis		
					OR	95% CI	*P* value
Age, years [a]		80.5 (75.5–88.3)	87 (80.3–90)	0.08	—	—	—
Male sex		14 (53.9)	14 (38.9)	0.24	—	—	—
History of catheter insertion		17 (65.4)	23 (63.9)	0.9	—	—	—
Use of anticancer and immunosuppressive agents		5 (19.2)	6 (16.7)	0.79	—	—	—
Surgical history		7 (26.9)	9 (25.0)	0.86	—	—	—
Pitt bacteremia score [a]		2 (1–5.3)	1 (0–2)	0.07	—	—	—
Pitt bacteremia score ≥ 2		15 (57.7)	17 (47.2)	0.41	—	—	—
Charlson comorbidity index, median [a]		3 (1–5)	2 (1.3–3.8)	0.29	—	—	—
Charlson comorbidity index ≥ 3		16 (61.5)	17 (47.2)	0.26	—	—	—
White blood cell count, /μL [a]		10,600 (6350–13,700)	10,000 (6050–13,000)	0.63	—	—	—
C-reactive protein, mg/dL [a]		10.1 (5.3–15.9)	9.8 (4–19.1)	0.91	—	—	—
Creatinine, mg/dL [a]		1.13 (0.62–1.53)	1.06 (0.72–1.62)	0.58	—	—	—
Detection of multiple bacteria in blood cultures		3 (11.5)	4 (11.1)	1.0	—	—	—
Sources of bacteremia				0.052	—	—	—
	Urinary tract infection	8 (30.8)	25 (69.4)		—	—	—
	Biliary tract infection	11 (42.3)	7 (19.4)		—	—	—
	Respiratory tract infection	4 (15.4)	3 (8.3)		—	—	—
	Catheter-related blood stream infection	1 (3.9)	0 (0)		—	—	—
	Skin and soft tissue infection	1 (3.9)	0 (0)		—	—	—
	Pancreatic infection	0 (0)	1 (2.8)		—	—	—
	Unknown	1 (3.9)	0 (0)		—	—	—
Initial treatment with carbapenem		7 (26.9)	10 (27.8)	1.0	—	—	—
Number of days from blood culture submission to a positive blood culture result [a]		1 (1–3)	1 (1–2)	0.26	—	—	—
Blood culture identification 2 panel		5 (19.2)	17 (47.2)	0.023	2.4	0.23–23.6	0.46
Prospective audit and feedback		7 (26.9)	26 (72.2)	0.0004	14.5	1.6–131.9	0.017

Data are expressed as number (%). [a] median (interquartile range). CMZ, cefmetazole; OR, odds ratio; CI, confidence interval.
